# Genome reduction and potential metabolic complementation of the dual endosymbionts in the whitefly *Bemisia tabaci*

**DOI:** 10.1186/s12864-015-1379-6

**Published:** 2015-03-21

**Authors:** Qiong Rao, Pierre-Antoine Rollat-Farnier, Dan-Tong Zhu, Diego Santos-Garcia, Francisco J Silva, Andrés Moya, Amparo Latorre, Cecilia C Klein, Fabrice Vavre, Marie-France Sagot, Shu-Sheng Liu, Laurence Mouton, Xiao-Wei Wang

**Affiliations:** Ministry of Agriculture Key Laboratory of Agricultural Entomology, Institute of Insect Sciences, Zhejiang University, 310058 Hangzhou, China; Université de Lyon, Université Lyon1, Laboratoire de Biométrie et Biologie Evolutive, UMR CNRS 558, 69622 Villeurbanne, Cedex France; Inria Grenoble Rhône-Alpes, Grenoble, France; Institut Cavanilles de Biodiversitat i Biologia Evolutiva, Universitat de València, Valencia, Spain; Unidad Mixta de Investigación en Genómica y Salud FISABIO-Salud Pública and Universitat de València, Valencia, Spain; School of Agriculture and Food Science, Zhejiang A & F University, 311300 Lin’an, Zhejiang China

**Keywords:** Endosymbiont, Genome, *Hamiltonella*, *Portiera*, Whitefly, Metabolic complementation

## Abstract

**Background:**

The whitefly *Bemisia tabaci* is an important agricultural pest with global distribution. This phloem-sap feeder harbors a primary symbiont, “*Candidatus* Portiera aleyrodidarum”, which compensates for the deficient nutritional composition of its food sources, and a variety of secondary symbionts. Interestingly, all of these secondary symbionts are found in co-localization with the primary symbiont within the same bacteriocytes, which should favor the evolution of strong interactions between symbionts.

**Results:**

In this paper, we analyzed the genome sequences of the primary symbiont *Portiera* and of the secondary symbiont *Hamiltonella* in the *B. tabaci* Mediterranean (MED) species in order to gain insight into the metabolic role of each symbiont in the biology of their host. The genome sequences of the uncultured symbionts *Portiera* and *Hamiltonella* were obtained from one single bacteriocyte of MED *B. tabaci.* As already reported, the genome of *Portiera* is highly reduced (357 kb), but has kept a number of genes encoding most essential amino-acids and carotenoids. On the other hand, *Portiera* lacks almost all the genes involved in the synthesis of vitamins and cofactors. Moreover, some pathways are incomplete, notably those involved in the synthesis of some essential amino-acids. Interestingly, the genome of *Hamiltonella* revealed that this secondary symbiont can not only provide vitamins and cofactors, but also complete the missing steps of some of the pathways of *Portiera*. In addition, some critical amino-acid biosynthetic genes are missing in the two symbiotic genomes, but analysis of whitefly transcriptome suggests that the missing steps may be performed by the whitefly itself or its microbiota.

**Conclusions:**

These data suggest that *Portiera* and *Hamiltonella* are not only complementary but could also be mutually dependent to provide a full complement of nutrients to their host. Altogether, these results illustrate how functional redundancies can lead to gene losses in the genomes of the different symbiotic partners, reinforcing their inter-dependency.

**Electronic supplementary material:**

The online version of this article (doi:10.1186/s12864-015-1379-6) contains supplementary material, which is available to authorized users.

## Background

Interactions between endosymbionts and insects are widespread in nature [1,2], and are particularly important in members of the suborder Sternorrhyncha such as whiteflies, aphids, psyllids and mealybugs. The ability of these insects to use an unbalanced food source such as plant sap is allowed by the interaction with primary bacterial endosymbionts that complement the diet by providing their host with essential amino acids [3]. These primary endosymbionts are confined in specialized host cells, the bacteriocytes, and are strictly vertically transmitted [4], leading to a parallel evolution with their insect hosts for millions of years [5-7]. One evolutionary consequence of this lifestyle is an extreme genome reduction and degradation compared to free living relatives. This convergent evolution has been observed in primary symbionts of many insects, particularly in members of the suborder Sternorrhyncha, such as *Buchnera aphidicola* in aphids (from 422 to 655 kb) [8,9], *Carsonella ruddii* in psyllids (158-166 kb) [10], *Portiera aleyrodidarum* in whiteflies (281-358 kb) [11-13], as well as *Moranella endobia* (538 kb) and *Tremblaya princeps* (139-171 kb) in mealybugs [6,14,15].

Besides these primary symbionts, insects often harbor secondary symbionts that are not required for their host’s survival or reproduction. Nonetheless, they can have broad and important effects on the host biology and ecology, ranging from mutualism to reproductive manipulation, which allow them to spread and be maintained in host populations [16]. These secondary symbionts are both vertically and horizontally transmitted [17,18] and inhabit a variety of tissues, including bacteriocytes. As they are intracellular, secondary symbionts also show a pattern of genome reduction but to a lesser extent than primary symbionts. Part of their genome is devoted to the biosynthesis of vitamins and cofactors. For example, *Hamiltonella defensa* in aphids is able to supply all the essential vitamins except for thiamine (B1) and pantothenate (B5) [19].

Interestingly, recent advances have shown that these secondary or co-resident symbionts can complement the metabolic network of the primary symbionts. For example, the pair of endosymbionts *Carsonella eucalypti* (primary symbiont) and *Heteropsylla cubana* (secondary symbiont) in psyllids exhibits strict complementarity in the biosynthesis of tryptophan [10], as is also the case in the aphid *Cinara cedri* where *Serratia* complements lineage specific gene losses of the primary endosymbiont *Buchnera* [20]. Similarly, the primary endosymbiont *Sulcia muelleri* supplies most of the essential amino acids to its hosts while the remaining ones are provided by different co-resident symbionts: *Baumannia cicadellinicola* in sharpshooters, *Hodgkinia cicadicola* in cicadas, *Zinderia insecticola* in spittlebugs and *Nasuia deltocephalinicola* in leafhoppers [21-24]. Furthermore, “*Candidatus* Moranella endobia” and “*Candidatus* Tremblaya princeps” contribute to intermediate steps of the pathways for the synthesis of amino acids [14]. These insects must thus be seen as holobionts where the full community of organisms inhabiting a host must be taken into account to understand its phenotype, including its metabolic capabilities [25]. Interestingly, comparative genomics has revealed that these losses are generally specific to some lineages of primary endosymbionts, suggesting that the ancestor was performing the lost function. The most probable evolutionary scenario is as follows. Co-infection between the primary and the secondary symbiont preceded the function loss, generating a redundancy of some metabolic functions. As selection acts at the level of the holobiont, any loss of a metabolic function in only one symbiotic partner is neutral [26,27]. Such losses in essential functions might thus lead to the observed pattern of complementation and to the inter-dependency between the symbiotic partners.

One interesting case for investigating metabolic complementation within symbiotic communities is the whitefly *Bemisia tabaci* Gennadius (Homoptera: Aleyrodidae). This whitefly is a complex of at least 35 cryptic species that differ in many ecological respects, including their potential for causing damage. Some of them are serious destructive pests of agricultural, horticultural and ornamental crops with worldwide distribution [28-30]. This phloem-feeding insect harbors the primary bacterial symbiont “*Candidatus* Portiera aleyrodidarum” (Oceanospirillales, referred as “*Portiera*” in this study) that is located within bacteriocytes [4], as well as a variety of secondary symbionts [31]. *Portiera* is an ancient symbiont of whiteflies, acquired 100-200 million years ago [32]. The recent sequencing of two genomes of *Portiera* for the Mediterranean (MED, formerly referred to as the ‘Q’ biotype) species and two for the Middle East Asia Minor 1 (MEAM1, formerly referred to as the ‘B’ biotype) species of *B. tabaci* indicate that this symbiont not only synthesizes essential amino acids but could also protect its host against oxidative stress by supplying carotenoids [11,13]. Another remarkable feature of these genomes is their extreme reduction with a size ranging from 281 to 358 kb, which is intermediate between those observed for classical primary symbionts and the most reduced ones. Such reduction opens widely the possibility that secondary symbionts complement some metabolic functions of *Portiera*. In whiteflies, secondary symbionts have been reported to affect the life parameters of their host including virus transmission [14], resistance to natural enemies like parasitic wasps [33], heat stress [34] and insecticides [35]. Until now, at least seven secondary symbionts of whitefly have been reported in *B. tabaci*, including *Hamiltonella* (Enterobacteriaceae), *Arsenophonus* (Enterobacteriaceae), *Wolbachia* (Rickettsiales), *Rickettisia* (Rickettsiales), *Cardinium* (Bacteroidetes), *Fritschea* (Chlamydiales) and *Hemipteriphilus* (Rickettsiales) [36,37]. They have different patterns of localization but all of them share bacteriocytes with *Portiera.* These frequent infections with secondary symbionts in addition to *Portiera* make *B. tabaci* an interesting model to investigate metabolic complementation within symbiotic communities. Moreover, two of them, *Hamiltonella* and *Arsenophonus*, reach almost fixation when present in the insect populations, but they have never yet been found together within the same host individual [36]. In addition, *Hamiltonella* was recently found to provide fitness advantage under nutritional stress conditions, making it a good candidate for analyzing complementation in *B. tabaci* [38].

Among the *B. tabaci* species complex, the MEAM1 and the MED species are the most widespread and economically important. Over the last twenty years, they have spread rapidly to more than eighty countries over six continents, highlighting their ability to adapt to various environments, and caused serious economic damages worldwide [30,39]. While MEAM1 is notorious for its survivability under extreme conditions, MED is highly resistant to some classes of pesticides, especially neonicotinoids [40]. Interestingly, *Hamiltonella* has so far only been detected in these two species of *B. tabaci*.

The aim of the present study was to acquire, analyze and study the genome sequences of *Hamiltonella* and *Portiera*. Most often *B. tabaci* individuals harbor more than one secondary symbiont species, which can lead to complex interactions [41]. Here, we used a natural line belonging to the MED species that only harbors *Hamiltonella* in addition to *Portiera*. The data obtained provide insights into the dynamics and evolution of symbiont genomes in intracellular ecosystems, and the mechanisms involved in the interactions among the symbiotic partners, especially in terms of metabolic complementation. They confirm that complementation in whiteflies is an on-going process, and that the co-evolution of different symbiotic partners can lead to inter-dependency.

## Results and discussion

### Isolation of endosymbionts and DNA amplification

**Figure 1:**
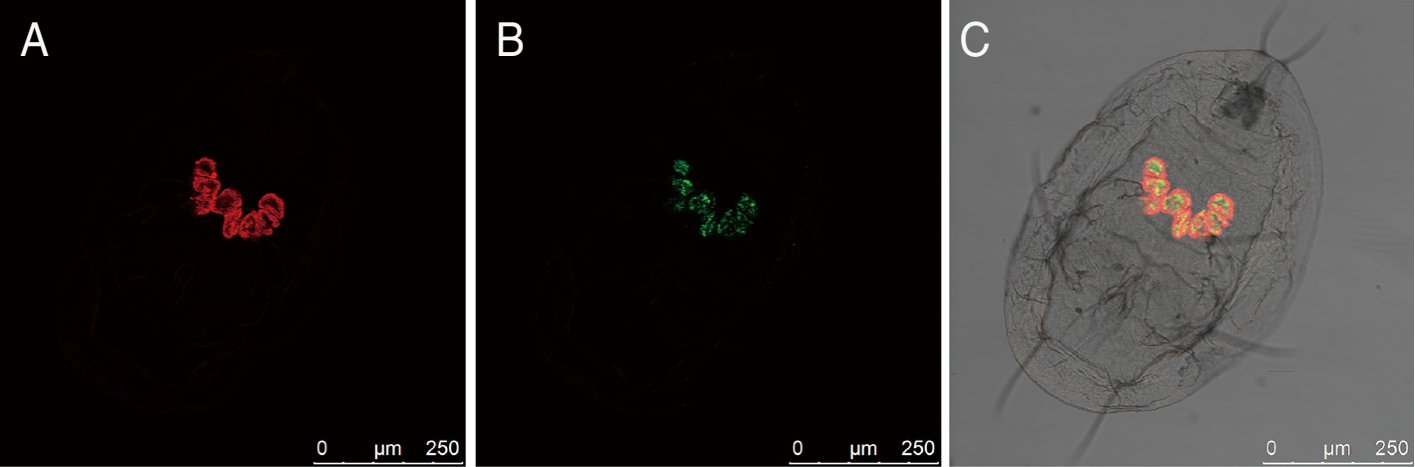
**Fluorescent** ***in situ*** **hybridization (FISH) of** ***B. tabaci*** **nymphs using** ***Portiera*** **(red) and** ***Hamiltonella*** **(green) specific probes. (A)** *Portiera* channel; **(B)** *Hamiltonella* channel; **(C)** *Portiera* and *Hamiltonella* channels on bright field channel, combined optical sections.

PCRs performed using specific primers of *Portiera*, *Hamiltonella*, *Cardinium*, *Wolbachia, Rickettsia, Arsenophonus* and *Hemipteriphilus* confirmed that the MED line used in this study only harbors *Portiera* and *Hamiltonella* (data not shown). In addition, fluorescence *in situ* hybridization (FISH) revealed that *Hamiltonella* shares bacteriocytes with *Porteria* in this line, and disperses in the cytoplasm of bacteriocytes (Figure 1), as already observed [31]. This contrasts with the situation observed in the aphid *Acyrthosiphon pisum* where *Hamiltonella,* is localized within sheath cells and do not share the same bacteriocytes with *Buchnera* [42,43]. This suggests that the two endosymbionts in MED whiteflies have a more intimate relationship. The endosymbionts were isolated by micromanipulation from a single bacteriocyte cell. Bacterial DNA was amplified by multiple displacement amplification. Diagnostic PCRs were then performed using specific primers to test for the presence of the endosymbiont DNA and the absence of host nuclear DNA contamination (Additional file 1: Figure S1).

### Sequencing and general features of the *Portiera* and *Hamitonella* genomes

Amplified symbiotic DNA was sequenced using the Illumina HiSeq 2000 sequencer and the genomes of both *Portiera* and *Hamiltonella* were independently assembled. The main features for these assemblies are summarized in Table 1. The genome of *Portiera* was assembled into a single contig. It has the classical characteristics of primary endosymbionts: a biased A + T content of 73.9% with a reduced genome (357,461 base pairs), but which does not reach the smaller sizes reported to date, such as for the genomes of *Nasuia deltocephalinic ola* (112 kb), *Tremblaya princeps* (139 kb), *Hodgkinia cicadicola* (144 kb), *Carsonella ruddii* (160 kb), *Zinderia insecticola* (209 kb) and *Sulcia muelleri* (246 kb), *Uzinura diaspidicola* (263 kb) (Table 2) [14,16,21,23,44-46]. Despite its reduced size, this genome has a relatively low percentage of coding DNA (67.4%), which is much less than the coding density of *Buchnera* in aphids (83%). Large intergenic regions have been shown to represent an important substrate for genome rearrangements in *Portiera* [12]. A total of 272 genes were predicted in the *Portiera* genome, and 84.5% of them (230 genes) have homologs present in GenBank. The genome encodes three rRNA genes (16S, 23S and 5S), two non-coding RNAs (rnpB, tmRNA) and 33 tRNA genes including at least one for each of the 20 amino acids (Table 3), as observed in the two other assembled *Portiera* genomes of the MED whitefly species and the two genomes of *Portiera* in the MEAM1 whitefly species [11,13,47]. The gene content is the same in all deposited genomes, the differences being only due to variations in the annotations and pseudogene detection parameters.

**Table 1:** **General statistics and features of** ***Portiera*** **and** ***Hamiltonella*** **genomes from the MED whitefly**

	***Portiera***	***Hamiltonella***
Total number of scaffold/contig	1	92
Current genome assembly (bp)	357,461	1,800,792
Average length (bp)	-	19,574
N50 length (bp)	-	102,662
N90 length (bp)	-	15,360
Maximum contig length (bp)	-	214,721
Predicted genes	272	1,884
Assigned function genes	230	1,672
Gene average length	889	806
Coding density, %	67.4	84.4
tRNA genes	33	40
rRNA genes	3 (5S, 16S, 23S)	3 (5S, 16S, 23S)
GC content, %	26.12	40.49

**Table 2:** **General genomic properties of representative free-living bacteria and insect symbionts**

	**Chromosome size, bp**	**G + C, %**	**No. of CDS**	**Coding density%**	**Avg. CDS length**	**Ref.**
**α-proteobacteria**						
*Rickettsia belli*	1,522,076	31.6	1,429	85.2	908	[78]
*Wolbachia pipientis*	1,267,782	35.2	1,195	80.2	851	[79]
*Hodgkinia cicadicola*	143,795	58.4	169	91.3	777	[45]
**Β-proteobacteria**						
*Profftella armatur*	459,399	24.2	366	88.0	1,104	[80]
*Zinderia insecticola*	208,564	13.5	202	89.7	926	[23]
*Tremblaya princeps*	138,927	58.8	121	66.1	759	[14]
*Nasuia deltocephalinicola*	112,091	17.1	137	91.9	752	[24]
**γ-proteobacteria**						
*Escherichia coli*	4,641,652	50.8	4,140	85.1	954	[81]
*Sodalis glossinidius*	4,171,146	54.7	2,432	50.9	873	[82]
*Arsenophonus nasoniae*	3,567,128^*^	37.4	3,332	-	-	[83]
*Hamiltonella defensa*	2,110,331^*^	40.1	2,094	80.4	810	[19]
	1,843,969	40.3	1,806	76.8	784	This study
*Serratia symbiotica*	1,762,765	29.2	672	38.8	1,019	[20]
*Blochmannia pennsylvanicus*	791,654	29.6	610	76.7	995	[84]
*Blochmannia floridanus*	705,557	27.4	583	83.2	1,007	[85]
*Baumannia cicadellinicola*	686,194	33.2	595	85.5	986	[21]
*Buchnera aphidicola*	640,681	26.3	564	87.0	988	[8]
	416,380	20.2	357	85.1	992	[9]
*Portiera aleyrodidarum*	357,461	26.1	272	67.7	890	This study
	357,472	26.1	246	67.7	984	[13]
	358,242	26.2	256	67.6	945	[11]
	280,663	24.7	269	94.3	984	[12]
*Carsonella ruddii*	159,662	16.6	182	94.1	826	[44]
**Bacteroidetes**						
*Uzinura diaspidicola*	263,431	30.2	227	86.5	1,004	[46]
*Sulcia muelleri*	245,530	22.4	227	92.1	996	[86]

*Uncompleted genome.

**Table 3:** **tRNA type of** ***Portiera*** **and** ***Hamiltonella*** **from the MED whitefly**

**tRNA type**	***Portiera*** **anti-codon**	***Hamiltonella*** **anti-codon**
Ala	TGC	TGC/GGC
Arg	TCT/ACG/CCG	TCT/CCT/CCG/ACG/TCT
Asn	GTT	GTT
Asp	GTC	GTC
Cys	GCA	GCA
Gln	TTG	CTG/TTG
Glu	TTC	TTC
Gly	GCC/TCC	TCC/GCC
His	GTG	GTG
Ile	GAT	GAT
kIle	CAT	CAT
Leu	TAG/GAG/TAA	CAG/GAG/TAG/TAA/CAA
Lys	TTT	TTT/CTT
iMet	CAT	CAT
Met	CAT	CAT
Phe	GAA	GAA
Pro	TGG	TGG/GGG
Ser	TGA/GCT/GGA/CGA	GCT/GGA/TGA
Thr	CGT/TGT/AGT	TGT/GGT
Trp	CCA	CCA
Tyr	GTA	GTA
Val	GAC/TAC	TAC/GAC

The draft genome of *Hamiltonella* in this MED line has an approximate size of 1,800,792 bp with an average G + C content of 40.49% and comprises 92 large scaffolds (Table 1) with a N50 size of approximately 102.66 kb. As only one bacteriocyte was used for bacterial DNA extraction and amplification, the incompleteness of the *Hamiltonella* genome is probably due to the bias during PCR. The genome encodes a total of 1,884 putative CDSs for which 88.75% (1,672 CDSs) can be assigned to a putative biological function. A total of 40 tRNA genes including at least one for all of the 20 amino acids and five small RNAs were predicted (Table 3). The average coding gene identity with *H. defensa* Hd-Ap is very high (approximately 96.4% average nucleotide similarity). Globally, the general features of this genome are very similar to those of other genomes of secondary symbionts.

### Functional annotation of the genomes of *Portiera* and *Hamiltonella* and metabolic reconstruction

Protein-coding genes of *Portiera* and *Hamiltonella* were classified into Clusters of Orthologous Groups (COGs) [48]. A total of 209 *Portiera* genes and 1,119 *Hamiltonella* genes were assigned to COGs (Additional file 2: Figure S2). The three most prominently represented COG categories in the *Portiera* genome are “Translation, ribosomal structure and biogenesis”, “Amino acid transport and metabolism”, and “Energy production and conversion” (COGs J, E, and C, respectively). These COG categories are essential for cellular metabolism of primary endosymbionts. Conversely, genes related to “RNA processing and modification”, “Chromatin structure and dynamics”, “Cell motility and Signal transduction mechanisms” (COGs A, B, N, and T, respectively) are absent from *Portiera*. In *Hamiltonella*, several COG categories are prominently represented, namely “Translation ribosomal structure and biogenesis”, “Replication, recombination and repair”, and “Cell wall/membrane/envelope biogenesis” (COGs J, L, and M, respectively) (Additional file 2: Figure S2).

The metabolic networks of the two symbionts were reconstructed and used to identify the sources needed by each symbiont (Tables 4 and 5). *Portiera* needs 11 different sources to produce less than 180 metabolites, while *Hamiltonella* needs 15 sources for less than 600 metabolites (Pearson test, p-value < 0.05), which conforms with the expectation of a more reduced and more host-dependent metabolism in this primary endosymbiont than in *Hamiltonella*. While most of these sources might be provided by the host, the metabolism of *Hamiltonella* needs some sources which would be only synthesized by *Portiera. AroA* is a 3-phosphoshikimate-1-carboxyvinyltransferase involved in the sixth step of the chorismate pathway, leading to the production of 5-enolpyruvyl-shikimate-3-phosphate. A mutation in the *aroA* gene, determined by the *in silico* analysis, and later confirmed by Sanger sequencing on independent samples (Additional file 3: Figure S3) suggests that 5-enolpyruvyl-shikimate-3-phosphate is a source for the *Hamiltonella* metabolism. *Portiera* can produce this source and probably export it to the secondary endosymbiont. Nevertheless, Tamas *et al.* [49] reported that a point mutation in a polyA tract can be rescued by transcriptional slippage and result in some functional protein. As the mutation of whitefly *aroA* gene is also present in the polyA tract (Additional file 3: Figure S3) and *aroA* is intact in the *Hamiltonella* from *A. pisum* [19], whether the whitefly *aroA* gene is functional or not warrants further investigation.

**Table 4:** **Necessary sources for the metabolism of** ***Portiera***

**Inputs**	**Class**	**Putative source**	**Proof**
(Seleno-)Homocysteine	Amino acid	*B. tabaci*	[5]
Geranylgeranyl diphosphate	Isoprenoid	*B. tabaci*	PWY-5120
HCO_3_	Anion	*B. tabaci*	[10]
L-Ornithine	Amino acid	*B. tabaci*	[20]
L-Aspartate	Amino acid	*B. tabaci* and *Hamiltonella*	ASPARTATESYN-PWY
Erythrose-4-phosphate	Sugar Phosphate	*B. tabaci* and *Hamiltonella*	NONOXIPENT-PWY
Phosphoenol-pyruvate	Carboxylic acid	*B. tabaci* and *Hamiltonella*	GLYCOLYSIS
Farnesyl-Diphosphate	Isoprenoid	*B. tabaci* and *Hamiltonella*	PWY-5123
Ribose-5-phosphate	Sugar Phosphate	*B. tabaci* and *Hamiltonella*	NONOXIPENT-PWY
Protoheme	Heme	Mitochondria	HEME-BIOSYNTHESIS-II

The sources potentially provided by *Hamiltonella* have been assessed from our analyses. Most of the sources not produced by the bacterial partner are classic metabolites of eukaryotes, and the corresponding biosynthetic pathways in MetaCyc are referred. Some sources do not seem to be produced by eukaryotes, but previous works on phloemophagous insects proposed that they were acquired from the host or its diet. The same assumptions have been made in this study, and the corresponding references have been indicated in the table.

**Table 5:** **Necessary sources for the metabolism of** ***Hamiltonella***

**Metabolite**	**Product/Pathway**	**Putative source**	**Proof**
H_2_S (S_2_O_3_)	(Seleno)Cysteine	*B. tabaci*	[87]
Pantothenate	Co-enzyme A	*B. tabaci*	[52]
HCO_3_	Fatty acids, nucleic acids etc.	*B. tabaci*	[10]
Dihydroneopterin	Folate	*B. tabaci*	[88]
P-Amino-Benzoate	Folate	*B. tabaci*	[89]
Glucose	Glucolysis	*B. tabaci*	[19]
Serine	Glycine, Cysteine etc.	*B. tabaci*	SERSYN-PWY
Fe^2+^	Heme o, general cofactor	*B. tabaci*	[89]
Proline	Glutamate	*B. tabaci*	PROSYN-PWY
E4P	Pyridoxine	*B. tabaci*	NONOXIPENT-PWY
SAM	Methionine	*B. tabaci*	PWY-5041
Protoporphyrin	Heme o	Mitochondria	HEME-BIOSYNTHESIS-II
5-ES-3P	Chorismate	*Portiera*	
N-S-LL-2,6-D	Lysine	*Portiera*	
Phenyl-pyruvate	Phenylalanine	*Portiera*	

The sources potentially providesssssd by *Portiera* have been assessed from our analyses. Most of the sources not produced by the bacterial partner are classic metabolites of eukaryotes, and the corresponding biosynthetic pathways in MetaCyc are referred. Some sources do not seem to be produced by eukaryotes, but previous works on phloemophagous insects proposed that they were acquired from the host or its diet. The same assumptions have been made in this study, and the corresponding references have been indicated in the table.

*Abbreviations*: D-erythrose-4-phosphate (E4P); S-adenosyl methionine (SAM); 5-enolpyruvyl-shikimate-3-phosphate (5-ES-3P); N-succinyl-L,L-2,6-diaminopimelate (N-S-LL-2,6-D).

**Figure 2:**
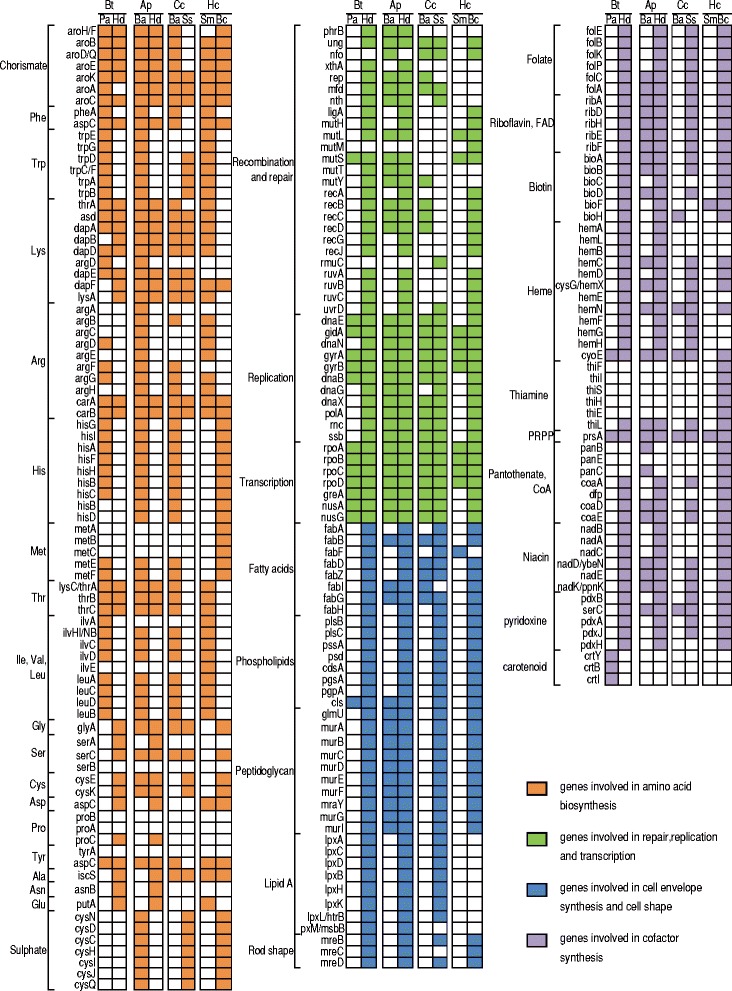
**Gene content of reduced genomes from symbionts** **[** 8 **,** 9 **,** 19 **-** 21 **,** 78 **].** Abbreviations : *Bt- Bemisia tabaci, Ap- Acyrthosiphon pisum, Cc- Cinara cedri, Hc- Homalodisca coagulata*; *Pa- Portiera aleyrodidarum, Hd- Hamiltonella defensa, Ba- Buchnera aphidicola, Ss- Serratia symbiotica, Sm- Sulcia muelleri, Bc- Baumannia cicadellinicola.*

Among sources provided by the host, hydrogen sulfide is of particular interest. Indeed, while the two symbionts of the MED whitefly lack the whole gene sets for sulfur metabolism (Figure 2), *cysN/cysC* [bifunctional enzyme EC: 2.7.7.4, 2.7.1.25], *cysQ* [EC: 3.1.3.7] and *cysJ* [EC: 1.8.1.2] of this pathway have been identified in the whitefly transcriptome [50]. It suggests that the host whitefly or its gut microbiota might help the symbionts for sulfate reduction. Notably, the sulfate reduction pathway can transform sulfate into hydrogen sulfide, a precursor for the cysteine synthesis pathway present in *Hamiltonella* (Table 5).

### Essential amino acid synthesis depends on within-pathway complementation between symbionts

A major metabolic contribution of symbionts in phloemophagous insects concerns the synthesis of essential amino acids. As highlighted by the COG analysis, some metabolic functions related to the biosynthesis of amino-acids have been retained in the highly reduced genome of *Portiera*, which contains 56 genes dedicated to this function (Additional file 4: Figure S4, Additional file 5: Table S1). *Portiera* is therefore able to synthesize or to participate in the synthesis of several amino acids, and especially essential ones, like other primary endosymbionts (Additional file 4: Figure S4). These results further demonstrate the evolutionary convergence between symbionts from distinct phylogenetic lineages hosted by hosts with similar diets [1,51].

However, *Portiera* has only two complete pathways for the synthesis of essential amino-acids (threonine and tryptophan) [11,13]. Interestingly, some of the missing genes in these pathways are present in the *Hamiltonella* genome (Figure 2). For example, *aspC*, a gene encoding an enzyme required for the phenylalanine synthesis, is missing in the genome of *Portiera* but is encoded by *Hamiltonella*. Furthermore, the genome of *Portiera* lacks three enzymes involved in the biosynthesis of lysine, namely *dapF*, *lysA*, which are absent, and *dapB* which is pseudogenized, while *Hamiltonella* possesses all of these genes but lacks the *argD* gene in the same pathway (Figure 2). As for chorismate in *Hamiltonella*, the production of lysine has only been recently lost in *Portiera*, since the entire pathway is present and intact in the *Portiera* of *Trialeurodes vaporariorum*, another member of Aleyrodidae [12]. This is consistent with the hypothesis that acquisition of secondary endosymbionts generates metabolic redundancies with the primary endosymbiont, leading to specific gene losses in both genomes [10,20]. These losses, if they affect essential functions ancestrally performed by the primary endosymbiont, can rapidly make secondary endosymbionts indispensable for the holobiont [10,20]. Nevertheless, the functions of *aspC* in *Portiera* and *argD* in *Hamiltonella* could also be performed by other aminotransferases encoded by their respective genomes, an unknown enzyme, or by an already known enzyme which would have changed or extended its prior function, as has already been proposed for other symbiotic genes [10,52]. Interestingly, the lysine (in *Carsonella, Sulcia* and some *Buchnera*) and phenylalanine (in *Carsonella*, *Buchnera*, *Tremblaya* and *Ishikawella*) pathways are often incomplete in the genomes of primary endosymbionts, and have been proposed to be in some cases complemented by the host or co-symbiont (reviewed in Hansen *et al*. [53]).

The host itself could also provide enzymes for completing some pathways, as has been recently shown in aphids where the host metabolism has been redirected to complement some missing reactions for amino acid synthesis in *Buchnera* [54], similar to what has been hypothesized to occur in the mealybugs and psyllids [6,7]. For example, it has been hypothesized that phloem-feeder hosts encode a homolog of the *aspC* gene allowing the production of phenylalanine [53]. The same situation might apply to *B. tabaci* MED for the synthesis of valine, leucine and isoleucine, for which *ilvE*, the gene encoding the common last enzyme of these pathways is absent from the genomes of both *Portiera* and *Hamiltonella* and has been found in the transcriptome of the MED whitefly [50].

The case of the histidine synthesis pathway is different because the genes which are not present in the genome of *Portiera* (*hisB* [EC 3.1.3.15] and *hisD*; [11,13]) are neither present in the *Hamiltonella*’s genome (Figure 2), nor have been detected in the whitefly’s transcriptome [50]. This suggests that histidine may be provided by the food. This hypothesis is relevant since previous studies indicated that this essential amino acid is present in high concentration in the phloem sap [55]. Finally, some genes involved in the synthesis of non-essential amino acids like serine and proline are missing in the genomes of both endosymbionts, but can be identified in the sequences obtained from the MED transcriptome [50]. This highlights the fact that gene losses in the symbiotic genomes can also occur when some functional redundancies exist with the host’s genome.

### *Portiera* and *Hamiltonella* synthesize different vitamins and cofactors

Other types of metabolites frequently provided by the symbionts in sap-feeding insects are vitamins and cofactors. *Portiera* lacks almost all the genes involved in the synthesis of vitamins and cofactors (Figure 2). However, *Portiera* is the first symbiont reported to be able to produce carotenoids [11,13]. Interestingly, a thorough BLAST search of the whitefly transcriptome [50] suggested that the insect cannot synthesize carotenoids, unlike aphids [56]. Conversely, *Hamiltonella* possesses a number of gene sets (76 genes) dedicated to vitamin production, which include riboflavin (vitamin B2), NAD, pyridoxine (vitamin B6), biotin (vitamin B7), folic acid (vitamin B9) (Figure 2, Additional file 4: Figure S4, Additional file 5: Table S2). However, it is unable to produce thiamine (vitamin B1) and pantothenic acid (vitamin B5) and, in the MED transcriptome, we did not find any genes except *panC* for synthesizing the two missing vitamins, suggesting that whiteflies might capture thiamine and pantothenic acid from the phloem sap. Because the MED transcriptome was sequenced using the whole whitefly [50], whether the *panC* gene is laterally acquired by the whitefly or produced by other symbionts (bacteria) within the whitefly is still unknown.

### Transport capabilities in link with exchanged metabolites

As revealed by previous analyses, the metabolic networks of both endosymbionts could be highly intertwined requiring efficient transport systems of metabolites. Compared to secondary symbionts, primary symbionts lack many genes for environmental information processing, such as membrane transport and signal transduction (Additional file 5: Table S2). In addition, most primary endosymbionts have kept only a few transporter systems although metabolite fluxes are required within the symbiotic system [57]. Indeed, the metabolism of *Portiera* needs amino acid, nucleic acid and isoprenoid uptake (Table 4). Moreover, the products of the metabolism of *Portiera* (carotenoids, essential amino acids) need also to be exported to the host.

However, the genome of *Portiera* encodes only eleven putative inner-membrane-located transporters (Additional file 5: Table S3). As the gene content of *Portiera* has quite not changed since the last ancestor between the obligate endosymbionts of *B. tabaci* and *T. vaporarium* [12], this suggests an ancient loss of transporters. The low number of transporters is of the same order of magnitude as in *Buchnera* Cc (12 transporters), the symbiont of *Cinara cedri*, and less than half of the number of transporters identified in other *Buchnera* genomes (30 to 34) [52]. Different hypotheses have been proposed to explain the weak number of transporters in *Buchnera* Cc [52]. First, this could be linked to the strong specialization of the aphid host of this symbiont (only found in cedar plants), but in our case, the large plant spectrum of *B. tabaci* is not consistent with this hypothesis. Second, the presence of general transporters with low affinity with the substrates, or the recruitment of transporters from the host, could compensate the losses. For example, aphid GLNT1 which transports glutamine is located in the bacteriocyte membrane instead of the symbiosomal membrane [58].

In addition, this reduction of the ancestral transporter stock can be explained by the small number of classes of chemical compounds transported by *Portiera*. Its genome encodes exporters/importers for all of these classes, excepted sugars, but as it is expected in *Buchnera* Cc, *Portiera* probably acquires them by passive diffusion [9]. For example, it encodes a *gltP* proton dependent transporter involved in *Escherichia coli* in the uptake of aspartate [59], a probable source of the *Portiera* metabolism. In addition, this transporter could also allow the uptake of the other required amino acids (Table 4). Another transporter is *argO*, which is normally involved in arginine export [60]. Interestingly, arginine is no more synthesized by *Portiera* from *B. tabaci*, and consequently, *argO* could have no more roles, or could be involved in the export of other essential amino acids synthesized by the endosymbiont. Finally, the best hits of BTQP_154 (>50% of homology) are *ditE*-like genes of *Pseudomonas spp*. The *ditE* permease could be involved in diterpenoid transport [61]. Thus, this transporter could be a putative candidate for the uptake of isoprenoid sources (Table 4) and the export of carotenoids.

## Conclusions

The analysis of the metabolic pathways in *Portiera* and *Hamiltonella* revealed the interdependency between two partners engaged in a mutualistic relationship. First, while the two partners depend principally on sources coming from the host diet and metabolism, they could also rely on some sources they provide to each other. Second, although *Portiera* encodes a large number of genes in the biosynthesis of essential amino-acids, it lacks almost all genes required for the synthesis of cofactors, while the latter are likely to be produced by *Hamiltonella*. Third, and in addition to this complementarity in terms of provided-sources and metabolites, some strict within-pathway complementation cases have been inferred by our *in silico* analysis, which concern two essential amino acids (lysine and phenylalanine). These results suggest that *Hamiltonella* could be a primary partner of the consortium, and could functionally explain the recent results showing the benefit it provides under nutritional stress conditions [38], and allow its fixation within populations. Interestingly, *Hamiltonella* probably replaced the primary endosymbiont for some functions that are still assumed by *Portiera* in other species of *B. tabaci.* As *Hamiltonella* is probably a recent symbiont of *Bemisia*, it appears that both symbionts have been caught in the act of a switch towards reciprocal complementation. Emergence of dependence between the symbiotic partners is rather due to metabolic redundancies which have been eliminated, a phenomenon that led to an apparent mutualism. Nevertheless, all of these postulations are based on the analysis of *Portiera* and *Hamiltonella* draft genomes. In order to confirm this scenario and gain insights into the evolutionary dynamics and history of these interactions, this analysis should be extended to other populations of MED species associated with *Arsenophonus*, and to other species of *B. tabaci*. This could allow determining when and in which *B. tabaci* species *Portiera* lost some key elements for the holobiont functioning, and whether some secondary endosymbionts only replaced these functions, or widened the initial niche of the holobiont, making it less dependent on specific environmental sources for some metabolites.

## Methods

### Whitefly rearing

The *B. tabaci* line belonging to the MED species (mtCO1 GenBank accession no: DQ473394) was maintained on cotton *Gossypium hirsutum* (Malvaceae) (cv. Zhe-Mian 1793) in climate chambers at 27 ± 1°C, 14 h : 10 h (light: darkness) and 40-60% relative humidity. The purity of this whitefly line was monitored every 3-5 generations using the RAPD-PCR technique with the primer H_16_ (5′-TCTCAGCTGG-3′) [62]. Our previous study confirmed this line only harbors two symbionts, the primary symbiont *Portiera* and the secondary symbiont *Hamiltonella* [36].

### Fluorescence *in situ* hybridization (FISH)

The symbiont localization was determined by performing FISH on nymphs as previously described [31,63] with symbiont-specific 16S/23S rRNA DNA probes: BTP1-Cy3 (TGTCAGTGTCAGCCCAGAAG) for *Portiera* and BTH-Cy5 (CCAGATTCCCAGACTTTACTCA) for *Hamiltonella*. The *Portiera*-specific probe was used for all the individuals for control. Stained samples were whole mounted and photographed on a confocal microscope (Leica). Specificity of detection was confirmed using no probe staining and RNase-digested specimen.

### Purification and amplification of endosymbionts DNA from a single bacteriocyte

MED whitefly nymphs with paired, roundish and orange color bacteriome were dissected in PBS (GIBCO®, Invitrogen, USA). A single bacteriocyte was collected with a micropipette on a glass slice and symbiont cells were then isolated by Eppendorf® microcapillary using a TransferMan® NK2 micromanipulator with a CellTram Vario (Eppendorf, Germany) under an inverted microscope. To prepare the total DNA of the symbionts, the symbiont cells in PBS were amplified by multiple displacement amplification (MDA) using the Repli-g UltraFast Mini Kit (Qiagen, Germany) according to the manufacturer’s protocol, with some modifications [64]. The yield of amplified total DNA was about 300 ng per microliter.

### Diagnostic PCR

The amplified DNA was checked by diagnostic PCR using primers specific for *Portiera* and *Hamiltonella* [65]. Contamination by DNA from *B. tabaci* was also checked by performing PCR on β*-*actin gene and EF1 gene. The β*-*actin gene primers were Actin-F (GCTGCCTCCACCTCATTAGA) and Actin-R (AGGGCGGTGATTTCCTTCT). The PCR parameters for β*-*Actin gene of *B. tabaci* were 4 min at 94°C, followed by 30 cycles of: 45 s at 94°C, 45 s at 60°C, 1 min at 72°C, and a final extension of 10 min at 72°C. The EF1 gene primers were EF-F (CGTCCCCATTTCTGGATGGCACGG) and EF-R (CATCTCGACGGACTTAACTTCAGT), and the PCR parameters for EF1 gene are described in Ghanim *et al*. [66].

### Genome sequencing, assembly and annotation

Using the amplified endosymbiotic DNA, two paired-end libraries with an average insert size of 200 bp and 2 kb were generated and sequenced respectively, using the Illumina HiSeq 2000 sequencer according to the manufacturer’s protocols in Beijing Genomics Institute, BGI (Shenzhen, China). The reads belonging to *Portiera* were retrieved based on previous published *Portiera* genomes and then assembled using SOAPdenovo v1.05 (http://soap.genomics.org.cn/). The *Portiera* genome was assembled in a single circular closed contig. The rest of the reads were used for *Hamiltonella* assembly. By testing a range of k-mers (21, 25, 33, 37, 47, 53 and 57), we selected 33-mer as final K-mer size for assembly with default parameters. The resulted contigs were connected according to the 200 bp and 2 kb mate-paired relationships. This assembly generated a draft genome of *Hamiltonella.* It has an approximate size of 1,800,792 bp and comprises 92 large scaffolds. The incompleteness of the *Hamiltonella* genome is probably due to the bias during PCR amplification. CDSs were then predicted by Glimmer v3.0, and a homologous comparison to a nonredundant public database was performed by BLAST for function annotation. Genes were assumed to be pseudogenes if they underwent more than 20% of reduction compared to orthologs following [67]. The annotation of COGs, gene ontologies, and EC numbers was performed using SWISSPROT. We used RNAmmer and tRNAscan to identify rRNAs [68] and tRNAs [69]. tRNA genes with anticodon CAT were discriminated according to Silva *et al*. [70].

### KEGG (Kyoto Encyclopedia of Genes and Genome) analysis

For functional categorization, the protein sequences of the symbionts were annotated by mapping to KEGG pathways. All genes of *Portiera* and *Hamiltonella* were analyzed based on BBH (bi-directional best hit) by using the KAAS tool [71], which compared the metabolic capacity of the two bacteria with the other primary and secondary symbionts (see Additional file 5: Table S2).

### Metabolic complementation analysis

PathwayTools [72] was used to build the metabolic networks of both *Portiera* and *Hamiltonella* on the basis of E.C. numbers and annotations. MetExplore [73] was used to check and curate each metabolic reconstruction individually. Cytoscape was used for network visualisation [74]. Each reaction removed or corrected in MetExplore was followed by a strict manual control (see below). The refined networks were used to identify the metabolic sources in the two symbionts, *i.e.* the inputs which permit to produce all the different metabolites. Thus, it was important to avoid the detection of sources with no biological sense. To do this, we removed reactions which were unlikely to take place in these organisms, or which did not interest us in the case of this work. More precisely, the automatic reconstruction generated isolated reactions which do not use any inputs produced by the metabolism of either symbiont (Additional file 5: Table S4 and S5). These reactions can correspond to spontaneous reactions that have been placed in the networks because they do not need enzymes and can thus theoretically occur in all organisms. They can also correspond to enzymes that can theoretically perform different reactions. In that case only the reactions that are connected with the rest of the network were kept. Some reactions can also involve generic metabolites (“a sugar”) and were replaced by the specific metabolites (“glucose”), or removed if these specific reactions were not connected to the rest of the network. Finally, reactions that are not assumed to occur in prokaryotes were also removed. For each removed reaction in the metabolic network of a given endosymbiont, we checked that the concerned metabolites were not a product or a source of the metabolism of the other endosymbiont. Finally, to detect sources, we applied the Borenstein’s method [75] on the two metabolic reconstructions independently, using the Igraph package (R software) [76]. Some sources were due to missing reactions in the pathways. It is the case for lysine or folate biosynthesis in *Hamiltonella*. In order to check for the validity of these sources, the missing genes were searched on the basis of both MetaCyc and KEGG maps. For each missing reaction, we used queries based on Blast, domain identification, and enzyme name/E.C number to identify putative candidates – the last two steps were performed using the MaGe platform.

### *Portiera* transporter analysis

Analysis of the transporters of *Portiera* was performed as described in Charles *et al*. [52]*.* At first, Blast was used against the Transport Classification DataBase (TCDB) in order to find a set of potential transporters and to assess their classification according to the TCDB. Then GO annotations of the homologous genes present in UniprotKB were used to identify genes involved in transport. The genes found from these two steps were manually curated. The enzymatic reactions generally need some cofactors to occur. These last are not produced by *Portiera* and have also to be transported inside the cell from the environment. Thus, we considered these cofactors as an additional source of the metabolism of *Portiera* (Additional file 5: Table S6). The Uniprot database allowed us to find the cofactors associated with the different enzymes encoded by *the Portiera* genome.

### Nucleotide sequence accession numbers

The complete genome sequence of “*Candidatus* Portiera aleyrodidarum” from the MED whiteflies has been deposited at GenBank under the accession number CP007563. The draft genome of “*Candidatus* Hamiltonella defense” from the MED whiteflies has been deposited under the accession number AJLH00000000 [77]. The version described in this paper is the second version, AJLH02000000.

## Additional files

Additional file 1: Figure S1. Amplified DNA and diagnostic PCR. Diagnostic PCRs were performed with *Portiera*-specific primers, *Hamiltonella*-specific primers and *B. tabaci* primers targeting *β-actin* and elongation factor gene (EF1). From left to right, S1 and S2-two samples of amplified bacterial DNA, B- amplified DNA from one whole bacteriocyte (including symbionts and the bacteriocyte of *B. tabaci*), A- DNA from adults of *B. tabaci*, N- DNA from nymphs of *B. tabaci,* C- negative control. Additional file 2: Figure S2. COG-based characterization of all proteins with annotated functions in *Portiera* and *Hamiltonella*. Additional file 3: Figure S3. Mutation in the *aroA* gene of *Hamiltonella* from whitefly and aphid. Ap: partial *aroA* CDS of *Hamiltonella* from aphid (GenBank: CP001277.1; 267 bp: 683, 503-683, 769); Bt: partial *aroA* CDS in this study (Q-B.HGL0000044); W1-W7: partial *aroA* CDS of *Hamiltonella* from adults of *B. tabaci* by PCR sequencing. Additional file 4: Figure S4. Comparative analysis of genes by metabolism functional KEGG categories from the genomes of *Portiera* and other bacteria. For abbreviations of symbionts, Tp: *Tremblaya princeps*, Hc: *Hodgkinia cicadicola*, Cr: *Carsonella ruddii*, Sm: *Sulcia muelleri*, Pa: *Portiera aleyrodidarum*, Ba-Cc : *Buchnera aphidicola* (*Cinara cedri*), Ba-Ap: *Buchnera aphidicola* (*Acyrthosiphon pisum*). Additional file 5: Table S1. Amino acid metabolism functional KEGG categories from the genomes of *Portiera.* **Table S2.** Comparative analysis of genes by functional KEGG categories. **Table S3.** Transporters. **Table S4.** Removed reactions in the metabolic network of *Portiera*. Legend: G(eneric reactions), I(solated reactions), P(seudogenes), Sp(ontaneous reactions), T(axonomic issues). **Table S5.** Reactions in the metabolic network of *Portiera*. Legend: G(eneric reactions), I(solated reactions), P(seudogenes), Sp(ontaneous reactions), T(axonomic issues). **Table S6.** Necessary cofactors for the *Portiera* enzymes. The left column gives the list of the cofactors (based on Uniprot information), the right column the number of associated enzymes.

## References

[1] Moran NA, McCutcheon JP, Nakabachi A (2008). Genomics and evolution of heritable bacterial symbionts. Annu Rev Genet.

[2] Moya A, Pereto J, Gil R, Latorre A (2008). Learning how to live together: genomic insights into prokaryote-animal symbioses. Nat Rev Genet.

[3] Douglas AE (1998). Nutritional interactions in insect-microbial symbioses: aphids and their symbiotic bacteria *Buchnera*. Annu Rev Entomol.

[4] Thao ML, Baumann P (2004). Evolutionary relationships of primary prokaryotic endosymbionts of whiteflies and their hosts. Appl Environ Microbiol.

[5] Wilson ACC, Ashton PD, Calevro F, Charles H, Colella S, Febvay G (2010). Genomic insight into the amino acid relations of the pea aphid, *Acyrthosiphon pisum*, with its symbiotic bacterium *Buchnera aphidicola*. Insect Mol Biol.

[6] Husnik F, Nikoh N, Koga R, Ross L, Duncan RP, Fujie M (2013). Horizontal gene transfer from diverse bacteria to an insect genome enables a tripartite nested mealybug symbiosis. Cell.

[7] Sloan DB, Nakabachi A, Richards S, Qu J, Murali SC, Gibbs RA (2014). Parallel histories of horizontal gene transfer facilitated extreme reduction of endosymbiont genomes in sap-feeding insects. Mol Biol Evol.

[8] Shigenobu S, Watanabe H, Hattori M, Sakaki Y, Ishikawa H (2000). Genome sequence of the endocellular bacterial symbiont of aphids *Buchnera* sp.. APS Nature.

[9] Perez-Brocal V, Gil R, Ramos S, Lamelas A, Postigo M, Michelena JM (2006). A small microbial genome: the end of a long symbiotic relationship?. Science.

[10] Sloan DB, Moran NA (2012). Genome reduction and co-evolution between the primary and secondary bacterial symbionts of psyllids. Mol Biol Evol.

[11] Sloan DB, Moran NA (2012). Endosymbiotic bacteria as a source of carotenoids in whiteflies. Biol Lett.

[12] Sloan DB, Moran NA (2013). The evolution of genomic instability in the obligate endosymbionts of whiteflies. Genome Biol Evol.

[13] Santos-Garcia D, Farnier PA, Beitia F, Zchori-Fein E, Vavre F, Mouton L (2012). Complete genome sequence of “*Candidatus* Portiera aleyrodidarum” BT-QVLC, an obligate symbiont that supplies amino acids and carotenoids to *Bemisia tabaci*. J Bacteriol.

[14] McCutcheon JP, von Dohlen CD (2011). An interdependent metabolic patchwork in the nested symbiosis of mealybugs. Curr Biol.

[15] Lopez-Madrigal S, Latorre A, Porcar M, Moya A, Gil R (2013). Mealybugs nested endosymbiosis: going into the ‘matryoshka’ system in *Planococcus citri* in depth. BMC Microbiol.

[16] Zchori-Fein E, Bourtzis K (2012). Manipulative tenants: bacteria associated with arthropods.

[17] Sintupachee S, Milne JR, Poonchaisri S, Baimai V, Kittayapong P (2006). Closely related *Wolbachia* strains within the pumpkin arthropod community and the potential for horizontal transmission via the plant. Microb Ecol.

[18] Chiel E, Zchori-Fein E, Inbar M, Gottlieb Y, Adachi-Hagimori T, Kelly SE (2009). Almost there: transmission routes of bacterial symbionts between trophic levels. PLoS One.

[19] Degnan PH, Yu Y, Sisneros N, Wing RA, Moran NA (2009). *Hamiltonella defensa,* genome evolution of protective bacterial endosymbiont from pathogenic ancestors. Proc Natl Acad Sci U S A.

[20] Lamelas A, Gosalbes MJ, Manzano-Marin A, Pereto J, Moya A, Latorre A (2011). *Serratia symbiotica* from the aphid *Cinara cedri*: *a* missing link from facultative to obligate insect endosymbiont. PLoS Genet.

[21] Wu D, Daugherty SC, Van Aken SE, Pai GH, Watkins KL, Khouri H (2006). Metabolic complementarity and genomics of the dual bacterial symbiosis of sharpshooters. PLoS Biol.

[22] McCutcheon JP, McDonald BR, Moran NA (2009). Convergent evolution of metabolic roles in bacterial co-symbionts of insects. Proc Natl Acad Sci U S A.

[23] McCutcheon JP, Moran NA (2010). Functional convergence in reduced genomes of bacterial symbionts spanning 200 My of evolution. Genome Biol Evol.

[24] Bennett GM, Moran NA (2013). Small, smaller, smallest: the origins and evolution of ancient dual symbioses in a phloem-feeding insect. Genome Biol Evol.

[25] Ferrari J, Vavre F (2011). Bacterial symbionts in insects or the story of communities affecting communities. Proc R Soc B-Biol Sci.

[26] Vautrin E, Vavre F (2009). Interactions between vertically transmitted symbionts: cooperation or conflict?. Trends in Microbiol.

[27] Vautrin E, Genieys S, Charles S, Vavre F (2008). Do vertically transmitted symbionts co-existing in a single host compete or cooperate? A modelling approach. J Evolution Biol.

[28] Dinsdale A, Cook L, Riginos C, Buckley YM, Barro PD (2010). Refined global analysis of *Bemisia tabaci* (Hemiptera: Sternorrhyncha: Aleyrodoidea: Aleyrodidae) mitochondrial cytochrome oxidase 1 to identify species level genetic boundaries. Ann Entomol Soc Am.

[29] De Barro PJ, Liu SS, Boykin LM, Dinsdale AB (2011). *Bemisia tabaci*: A statement of species status. Annu Rev Entomol.

[30] Hu J, De Barro P, Zhao H, Wang J, Nardi F, Liu SS (2011). An extensive field survey combined with a phylogenetic analysis reveals rapid and widespread invasion of two alien whiteflies in China. PLoS One.

[31] Gottlieb Y, Ghanim M, Gueguen G, Kontsedalov S, Vavre F, Fleury F (2008). Inherited intracellular ecosystem: symbiotic bacteria share bacteriocytes in whiteflies. FASEB J.

[32] Baumann P (2005). Biology of bacteriocyte-associated endosymbionts of plant sap-sucking insects. Annu Rev Microbiol.

[33] Mahadav A, Gerling D, Gottlieb Y, Czosnek H, Ghanim M (2008). Parasitization by the wasp *Eretmocerus mundus* induces transcription of genes related to immune response and symbiotic bacteria proliferation in the whitefly *Bemisia tabaci*. BMC Genomics.

[34] Brumin M, Kontsedalov S, Ghanim M (2011). *Rickettsia* influences thermotolerance in the whitefly *Bemisia tabaci* B biotype. Insect Sci.

[35] Ghanim M, Kontsedalov S (2009). Susceptibility to insecticides in the Q biotype of *Bemisia tabaci* is correlated with bacterial symbiont densities. Pest Manag Sci.

[36] Bing XL, Ruan YM, Rao Q, Wang XW, Liu SS (2013). Diversity of secondary endosymbionts among different putative species of the whitefly *Bemisia tabaci*. Insect Sci.

[37] Bing XL, Yang J, Zchori-Fein E, Wang XW, Liu SS (2013). Characterization of a newly discovered symbiont of the whitefly *Bemisia tabaci* (Hemiptera: Aleyrodidae). Appl Environ Microbiol.

[38] Su Q, Xie W, Wang S, Wu Q, Liu B, Fang Y (2014). The endosymbiont *Hamiltonella* increases the growth rate of its host *Bemisia tabaci* during periods of nutritional stress. PLoS One.

[39] Rao Q, Luo C, Zhang H, Guo X, Devine GJ (2011). Distribution and dynamics of *Bemisia tabaci* invasive biotypes in central China. Bull Entomol Res.

[40] Rao Q, Xu YH, Luo C, Zhang HY, Jones CM, Devine GJ (2012). Characterisation of neonicotinoid and pymetrozine resistance in strains of *Bemisia tabaci* (Hemiptera: Aleyrodidae) from China. J Integr Agr.

[41] Gueguen G, Vavre F, Gnankine O, Peterschmitt M, Charif D, Chiel E (2010). Endosymbiont metacommunities, mtDNA diversity and the evolution of the *Bemisia tabaci* (Hemiptera: Aleyrodidae) species complex. Mol Ecol.

[42] Sandstrom JP, Russell JA, White JP, Moran NA (2001). Independent origins and horizontal transfer of bacterial symbionts of aphids. Mol Ecol.

[43] Moran NA, Russell JA, Koga R, Fukatsu T (2005). Evolutionary relationships of three new species of Enterobacteriaceae living as symbionts of aphids and other insects. Appl Environ Microbiol.

[44] Nakabachi A, Yamashita A, Toh H, Ishikawa H, Dunbar HE, Moran NA (2006). The 160-kilobase genome of the bacterial endosymbiont *Carsonella*. Science.

[45] McCutcheon JP, McDonald BR, Moran NA (2009). Origin of an alternative genetic code in the extremely small and GC-rich genome of a bacterial symbiont. PLoS Genet.

[46] Sabree ZL, Huang CY, Okusu A, Moran NA, Normark BB (2013). The nutrient supplying capabilities of *Uzinura*, an endosymbiont of armoured scale insects. Environ Microbiol.

[47] Jiang ZF, Xia F, Johnson KW, Bartom E, Tuteja JH, Stevens R (2012). Genome sequences of the primary endosymbiont “*Candidatus* Portiera aleyrodidarum” in the whitefly *Bemisia tabaci* B and Q biotypes. J Bacteriol.

[48] Tatusov RL, Koonin EV, Lipman DJ (1997). A genomic perspective on protein families. Science.

[49] Tamas I, Wernegreen JJ, Nystedt B, Kauppinen SN, Darby AC, Gomez-Valero L (2008). Endosymbiont gene functions impaired and rescued by polymerase infidelity at poly(A) tracts. Proc Natl Acad Sci U S A.

[50] Wang XW, Luan JB, Li JM, Bao YY, Zhang CX, Liu SS (2010). *De novo* characterization of a whitefly transcriptome and analysis of its gene expression during development. BMC Genomics.

[51] Lopez-Sanchez MJ, Neef A, Pereto J, Patino-Navarrete R, Pignatelli M, Latorre A (2009). Evolutionary convergence and nitrogen metabolism in *Blattabacterium* strain Bge, primary endosymbiont of the cockroach *Blattella germanica*. PLoS Genet.

[52] Charles H, Balmand S, Lamelas A, Cottret L, Perez-Brocal V, Burdin B (2011). A genomic reappraisal of symbiotic function in the aphid/*Buchnera* symbiosis: reduced transporter sets and variable membrane organisations. PLoS One.

[53] Hansen AK, Moran NA (2014). The impact of microbial symbionts on host plant utilization by herbivorous insects. Mol Ecol.

[54] Russell CW, Bouvaine S, Newell PD, Douglas AE (2013). Shared metabolic pathways in a coevolved insect-bacterial symbiosis. Appl Environ Microbiol.

[55] Douglas AE (2006). Phloem-sap feeding by animals: problems and solutions. J Exp Bot.

[56] Moran NA, Jarvik T (2010). Lateral transfer of genes from fungi underlies carotenoid production in aphids. Science.

[57] Ren Q, Paulsen IT (2005). Comparative analyses of fundamental differences in membrane transport capabilities in prokaryotes and eukaryotes. PLoS Comput Biol.

[58] Price DRG, Feng H, Baker JD, Bavan S, Luetje CW, Wilson ACC (2014). Aphid amino acid transporter regulates glutamine supply to intracellular bacterial symbionts. Proc Natl Acad Sci U S A.

[59] Wallace B, Yang YJ, Hong JS, Lum D (1990). Cloning and sequencing of a gene encoding a glutamate and aspartate carrier of *Escherichia coli* K-12. J Bacteriol.

[60] Nandineni MR, Gowrishankar J (2004). Evidence for an arginine exporter encoded by *ygg*A (*argO*) that is regulated by the *LysR*-type transcriptional regulator *ArgP* in *Escherichia coli*. J Bacteriol.

[61] Martin VJ, Mohn WW (2000). Genetic investigation of the catabolic pathway for degradation of abietane diterpenoids by *Pseudomonas abietaniphila* BKME-9. J Bacteriol.

[62] De Barro PJ, Driver F (1997). Use of RAPD-PCR to distinguish the B biotype from other biotypes of *Bemisia tabaci* (Gennadius) (Hemiptera: Aleyrodidae). Aust J Entomol.

[63] Gottlieb Y, Ghanim M, Chiel E, Gerling D, Portnoy V, Steinberg S (2006). Identification and localization of a *Rickettsia* sp. in *Bemisia tabaci* (Homoptera : Aleyrodidae). Appl Environ Microbiol.

[64] Woyke T, Tighe D, Mavromatis K, Clum A, Copeland A, Schackwitz W (2010). One bacterial cell, one complete genome. PLoS One.

[65] Zchori-Fein E, Brown JK (2002). Diversity of prokaryotes associated with *Bemisia tabaci* (Gennadius) (Hemiptera : Aleyrodidae). Ann Entomol Soc Am.

[66] Ghanim M, Sobol I, Ghanim M, Czosnek H (2007). Horizontal transmission of begomoviruses between *Bemisia tabaci* biotypes. Arthropod-Plant Interactions.

[67] Lerat E, Ochman H (2005). Recognizing the pseudogenes in bacterial genomes. Nucleic Acids Res.

[68] Lagesen K, Hallin P, Rodland EA, Staerfeldt HH, Rognes T, Ussery DW (2007). Consistent and rapid annotation of ribosomal RNA genes. Nucleic Acids Res.

[69] Schattner P, Brooks AN, Lowe TM (2005). The tRNAscan-SE, snoscan and snoGPS web servers for the detection of tRNAs and snoRNAs. Nucleic Acids Res.

[70] Silva FJ, Belda E, Talens SE (2006). Differential annotation of tRNA genes with anticodon CAT in bacterial genomes. Nucleic Acids Res.

[71] Moriya Y, Itoh M, Okuda S, Yoshizawa AC, Kanehisa M (2007). KAAS: an automatic genome annotation and pathway reconstruction server. Nucleic Acids Res.

[72] Karp PD, Paley S, Romero P (2002). The pathway tools software. Bioinformatics.

[73] Cottret L, Wildridge D, Vinson F, Barrett MP, Charles H, Sagot MF (2010). MetExplore: a web server to link metabolomic experiments and genome-scale metabolic networks. Nucleic Acids Res.

[74] Smoot ME, Ono K, Ruscheinski J, Wang PL, Ideker T (2011). Cytoscape 2.8: new features for data integration and network visualization. Bioinformatics.

[75] Borenstein E, Kupiec M, Feldman MW, Ruppin E (2008). Large-scale reconstruction and phylogenetic analysis of metabolic environments. Proc Natl Acad Sci U S A.

[76] Csardi G, Nepusz T (2012). The igraph software package for complex network research.

[77] Rao Q, Wang S, Su YL, Bing XL, Liu SS, Wang XW (2012). Draft genome sequence of “*Candidatus* Hamiltonella defensa”, an endosymbiont of the whitefly. Bemisia tabaci J Bacteriol.

[78] Ogata H, La Scola B, Audic S, Renesto P, Blanc G, Robert C, et al. Genome sequence of *Rickettsia bellii* illuminates the role of amoebae in gene exchanges between intracellular pathogens. PLoS Genet. 2006;2:e76. 10.1371/journal.pgen.0020076 PMC1458961 16703114

[79] Wu M, Sun LV, Vamathevan J, Riegler M, Deboy R, Brownlie JC, et al. Phylogenomics of the reproductive parasite *Wolbachia pipientis* wMel: a streamlined genome overrun by mobile genetic elements. PLoS Biol. 2004;2:e69. 10.1371/journal.pbio.0020069 PMC368164 15024419

[80] Nakabachi A, Ueoka R, Oshima K, Teta R, Mangoni A, Gurgui M, et al. Defensive bacteriome symbiont with a drastically reduced genome. Curr Biol. 2013;23:1478–84. 10.1016/j.cub.2013.06.027 23850282

[81] Blattner FR, Plunkett 3rd G, Bloch CA, Perna NT, Burland V, Riley M, et al. The complete genome sequence of *Escherichia coli* K-12. Science. 1997;277:1453–62. 10.1126/science.277.5331.1453 9278503

[82] Toh H, Weiss BL, Perkin SA, Yamashita A, Oshima K, Hattori M, et al. Massive genome erosion and functional adaptations provide insights into the symbiotic lifestyle of *Sodalis glossinidius* in the tsetse host. Genome Res. 2006;16:149–56. 10.1101/gr.4106106 PMC1361709 16365377

[83] Darby AC, Choi JH, Wilkes T, Hughes MA, Werren JH, Hurst GD, et al. Characteristics of the genome of *Arsenophonus nasoniae*, son-killer bacterium of the wasp *Nasonia*. Insect Mol Biol. 2010;19 Suppl 1:75–89. 10.1111/j.1365-2583.2009.00950.x 20167019

[84] Degnan PH, Lazarus AB, Wernegreen JJ. Genome sequence of *Blochmannia pennsylvanicus* indicates parallel evolutionary trends among bacterial mutualists of insects. Genome Res. 2005;15:1023–33. 10.1101/gr.3771305 PMC1182215 16077009

[85] Gil R, Silva FJ, Zientz E, Delmotte F, Gonzalez-Candelas F, Latorre A, et al. The genome sequence of *Blochmannia floridanus*: comparative analysis of reduced genomes. Proc Natl Acad Sci U S A. 2003;100:9388–93. 10.1073/pnas.1533499100 PMC170928 12886019

[86] McCutcheon JP, Moran NA. Parallel genomic evolution and metabolic interdependence in an ancient symbiosis. Proc Natl Acad Sci U S A. 2007;104:19392–7. 10.1073/pnas.0708855104 PMC2148300 18048332

[87] Zientz E, Dandekar T, Gross R (2004). Metabolic interdependence of obligate intracellular bacteria and their insect hosts. Microbiol Mol Biol Rev.

[88] Cottret L, Milreu PV, Acuña V, Marchetti-Spaccamela A, Stougie L, Charles H (2010). Graph-based analysis of the metabolic exchanges between two co-resident intracellular symbionts, *Baumannia cicadellinicola* and *Sulcia muelleri*, with their insect host, *Homalodisca coagulata*. PLoS Comput Biol.

[89] Lin H, Lou B, Glynn JM, Doddapaneni H, Civerolo EL, Chen C (2011). The complete genome sequence of “*Candidatus* Liberibacter solanacearum”, the bacterium associated with potato zebra chip disease. PLoS One.

