# Supplementary material for: Genome reduction and potential metabolic complementation of the dual endosymbionts in the whitefly Bemisia tabaci
Source: BMC Genomics. 2015 Mar 21;16(1):226. doi: 10.1186/s12864-015-1379-6 (PMC4438442; doi:10.1186/s12864-015-1379-6)
Supplement: Additional file 5: Table S1. — Amino acid metabolism functional KEGG categories from the genomes of Portiera. Table S2. Comparative analysis of genes by functional KEGG categories. Table S3. Transporters. Table S4. Removed reactions in the metabolic network of Portiera. Legend: G(eneric reactions), I(solated reactions), P(seudogenes), Sp(ontaneous reactions), T(axonomic issues). Table S5. Reactions in the metabolic network of Portiera. Legend: G(eneric reactions), I(solated reactions), P(seudogenes), Sp(ontaneous reactions), T(axonomic issues). Table S6. Necessary cofactors for the Portiera enzymes. The left column gives the list of the cofactors (based on Uniprot information), the right column the number of associated enzymes. [file 12864_2015_1379_MOESM5_ESM.doc]

**Table S1: Amino acid metabolism functional KEGG categories from the genomes of *Portiera***

| **The gene No. of *Portiera*** | **ko_ID** | **ko_name** |
| --- | --- | --- |
| BTQP_021 | K01754 | E4.3.1.19, ilvA, tdcB |
| BTQP_034 | K00611 | OTC, argF, argI |
| BTQP_035 | K01704 | leuD |
| BTQP_036 | K00052 | E1.1.1.85, leuB |
| BTQP_037 | K00133 | E1.2.1.11, asd |
| BTQP_039 | K01817 | trpF |
| BTQP_045 | K01687 | ilvD |
| BTQP_049 | K02502 | hisZ |
| BTQP_065 | K03043 | rpoB |
| BTQP_066 | K03046 | rpoC |
| BTQP_095 | K03040 | rpoA |
| BTQP_112 | K00164 | OGDH, sucA |
| BTQP_113 | K00658 | DLST, sucB |
| BTQP_114 | K00382 | DLD, lpd, pdhD |
| BTQP_130 | K00962 | pnp, PNPT1 |
| BTQP_135 | K11755 | hisI |
| BTQP_138 | K00163 | aceE |
| BTQP_139 | K01714 | dapA |
| BTQP_141 | K00800 | aroA |
| BTQP_142 | K14170 | pheA |
| BTQP_145 | K01703 | leuC |
| BTQP_146 | K01736 | E4.2.3.5, aroC |
| BTQP_149 | K01649 | E2.3.3.13, leuA |
| BTQP_155 | K01735 | aroB |
| BTQP_156 | K00891 | E2.7.1.71, aroK, aroL |
| BTQP_160 | K01523 | hisE |
| BTQP_161 | K00549 | E2.1.1.14, metE |
| BTQP_162 | K01626 | E2.5.1.54, aroF, aroG, aroH |
| BTQP_165 | K02337 | DPO3A1, dnaE |
| BTQP_169 | K00674 | dapD |
| BTQP_170 | K01439 | dapE |
| BTQP_175 | K01956 | carA, CPA1 |
| BTQP_176 | K01955 | carB, CPA2 |
| BTQP_192 | K03786 | aroQ, qutE |
| BTQP_202 | K00014 | aroE |
| BTQP_214 | K00928 | E2.7.2.4, lysC |
| BTQP_218 | K01696 | E4.2.1.20B, trpB |
| BTQP_219 | K01695 | trpA |
| BTQP_223 | K00821 | argD |
| BTQP_227 | K01940 | E6.3.4.5, argG |
| BTQP_231 | K01657 | trpE |
| BTQP_233 | K01658 | trpG |
| BTQP_234 | K00766 | trpD |
| BTQP_235 | K01609 | trpC |
| BTQP_246 | K00765 | E2.4.2.17, hisG |
| BTQP_254 | K02204 | E2.7.1.39B, thrB |
| BTQP_255 | K00817 | hisC |
| BTQP_256 | K02500 | hisF |
| BTQP_257 | K01814 | E5.3.1.16, hisA |
| BTQP_258 | K02501 | hisH |
| BTQP_259 | K01693 | E4.2.1.19, hisB |
| BTQP_263 | K01652 | E2.2.1.6L, ilvB, ilvG, ilvI |
| BTQP_264 | K01653 | E2.2.1.6S, ilvH, ilvN |
| BTQP_265 | K00053 | ilvC |
| BTQP_269 | K00003 | E1.1.1.3 |
| BTQP_270 | K01733 | E4.2.3.1, thrC |

**Table S2**: Comparative analysis of genes by functional KEGG categories.

| **KEGG Pathway Abbreviation*** | | ***Ec*** | ***Nd*** | ***Tp*** | ***Hc*** | ***Cr*** | ***Sm*** | ***Pa*** | ***Ba-Cc*** | ***Ba-Ap*** | ***Rb*** | ***Wp*** | ***Ss*** | ***Bc*** | ***Bf*** | ***Bp*** | ***Hd-Bt*** | ***Hd-Ap*** | ***An*** |
| --- | --- | --- | --- | --- | --- | --- | --- | --- | --- | --- | --- | --- | --- | --- | --- | --- | --- | --- | --- |
| Cellular Processes | Cell Growth and Death | 9 | 2 | 3 | 1 | 3 | 3 | 4 | 7 | 9 | 14 | 17 | 11 | 7 | 10 | 10 | 11 | 11 | 12 |
|  | Cell Motility | 47 | 0 | 0 | 0 | 0 | 0 | 0 | 8 | 21 | 0 | 0 | 0 | 0 | 0 | 0 | 0 | 0 | 37 |
|  | Transport and Catabolism | 6 | 0 | 0 | 0 | 1 | 0 | 0 | 0 | 1 | 2 | 3 | 1 | 1 | 1 | 1 | 2 | 2 | 6 |
| Environmental  Information  Processing | Membrane Transport | 148 | 0 | 0 | 0 | 0 | 3 | 6 | 6 | 18 | 34 | 33 | 35 | 28 | 25 | 25 | 85 | 85 | 92 |
|  | Signal Transduction | 101 | 0 | 0 | 3 | 0 | 2 | 0 | 3 | 5 | 18 | 16 | 7 | 4 | 5 | 4 | 24 | 25 | 60 |
| Genetic Information , Processing | Folding, Sorting and Degradation | 32 | 2 | 3 | 2 | 2 | 11 | 8 | 18 | 26 | 24 | 24 | 32 | 32 | 23 | 25 | 35 | 36 | 48 |
|  | Replication and Repair | 47 | 1 | 6 | 4 | 7 | 8 | 7 | 25 | 32 | 45 | 42 | 41 | 44 | 30 | 32 | 60 | 58 | 63 |
|  | Transcription | 1 | 0 | 3 | 3 | 3 | 3 | 3 | 3 | 3 | 4 | 2 | 3 | 4 | 3 | 4 | 4 | 4 | 4 |
|  | Translation | 62 | 29 | 27 | 35 | 30 | 46 | 62 | 71 | 80 | 78 | 72 | 81 | 80 | 76 | 78 | 78 | 82 | 82 |
| Metabolism | Amino Acid Metabolism | 179 | 7 | 33 | 18 | 32 | 55 | 56 | 57 | 90 | 56 | 64 | 62 | 73 | 95 | 97 | 86 | 84 | 144 |
|  | Biosynthesis of Other Secondary Metabolites | 16 | 0 | 0 | 1 | 0 | 1 | 2 | 2 | 2 | 4 | 3 | 2 | 3 | 5 | 5 | 3 | 3 | 8 |
|  | Carbohydrate Metabolism | 200 | 1 | 7 | 2 | 11 | 18 | 14 | 28 | 39 | 44 | 41 | 45 | 42 | 52 | 52 | 68 | 69 | 111 |
|  | Energy Metabolism | 104 | 5 | 2 | 13 | 10 | 11 | 21 | 35 | 53 | 59 | 61 | 49 | 67 | 67 | 66 | 66 | 66 | 92 |
|  | Enzyme Families | 8 | 0 | 0 | 0 | 0 | 0 | 0 | 0 | 1 | 0 | 2 | 3 | 2 | 4 | 4 | 2 | 2 | 6 |
|  | Glycan Biosynthesis and Metabolism | 48 | 0 | 0 | 0 | 1 | 0 | 2 | 2 | 20 | 30 | 18 | 36 | 18 | 31 | 33 | 43 | 41 | 52 |
|  | Lipid Metabolism | 58 | 0 | 0 | 4 | 0 | 1 | 1 | 6 | 14 | 19 | 18 | 30 | 32 | 33 | 33 | 26 | 27 | 47 |
|  | Metabolism of Cofactors and Vitamins | 107 | 2 | 6 | 9 | 6 | 11 | 10 | 16 | 42 | 38 | 46 | 52 | 66 | 51 | 55 | 76 | 75 | 111 |
|  | Metabolism of Other Amino Acids | 48 | 0 | 1 | 6 | 4 | 1 | 4 | 8 | 18 | 14 | 14 | 18 | 21 | 15 | 16 | 22 | 20 | 36 |
|  | Metabolism of Terpenoids and Polyketides | 40 | 0 | 0 | 0 | 1 | 3 | 2 | 2 | 11 | 14 | 18 | 15 | 15 | 19 | 24 | 25 | 25 | 37 |
|  | Nucleotide Metabolism | 80 | 6 | 7 | 7 | 7 | 9 | 8 | 18 | 44 | 32 | 46 | 54 | 55 | 44 | 46 | 56 | 55 | 73 |
|  | Xenobiotics Biodegradation and Metabolism | 44 | 0 | 0 | 1 | 0 | 1 | 1 | 1 | 2 | 7 | 8 | 3 | 4 | 14 | 14 | 8 | 8 | 21 |

***Abbreviation** -for abbreviations of free-living and insect symbionts, see Table 2

*An: Arsenophonus nasoniae*, Ba-Ap: Buchnera aphidicola-Ap, Ba-Cc: Buchnera aphidicola-Cc, Bc: Baumannia cicadellinicola, Bf: Blochmannia floridanus, Bp: Blochmannia pennsylvanicus, Cr: Carsonella ruddii, Ec: Escherichia coli, Hc: Hodgkinia cicadicola, Hd-Ap: Hamiltonella defensa-aphid, Hd-Bt: Hamiltonella defensa-whitefly, Nd: Nasuia deltocephalinicola, Pa-Bt: Portiera aleyrodidarum-whitefly, Rb: Rickettsia belli, Sm: Sulcia muelleri, Ss: Serratia symbiotica, Tp: Tremblaya princeps, Wg: , Wp: Wolbachia pipientis*

**Table S3:** Transporters

| **Transporter** | **Substrat** | **Import-Export** |
| --- | --- | --- |
| yidC | Proteins | Export |
| SEC translocon | Proteins | Export |
| argO | Amino Acids | Export |
| gltP | Aspartate (other amino acids?) | Import |
| mgtE | Magnesium (other cations?) | Import |
| F-ATP synthase | H+ | Import/Export |
| BTQP_154 (ditE-like) | Carotenoids? | Import/Export? |
| marC | Unknown | Unknown |
| yggT | Unknown | Unknown |
| BTQP_253 | Unknown | Unknown |
| BTQP_56/57 | Unknown | Unknown |

**Table S4: Removed reactions in the metabolic network of *Portiera*.** Legend: G(eneric reactions), I(solated reactions), P(seudogenes), Sp(ontaneous reactions), T(axonomic issues).

| **Removed reactions** | **Reasons** |
| --- | --- |
| 1.11.1.15-RXN | G ; I |
| 3.4.11.18-RXN | G ; I |
| 3.4.21.53-RXN | G ; I |
| 3.4.24.57-RXN | G ; I |
| 3.5.1.88-RXN | I |
| 5.99.1.3-RXN | G ; I |
| 6PGLUCONDEHYDROG-RXN | I |
| ACETYLORNTRANSAM-RXN | I |
| ALDOSE-1-EPIMERASE-RXN | I |
| ASPARTATEKIN-RXN | P |
| CARDIOLIPSYN-RXN | G ; I |
| DIHYDLIPACETRANS-RXN | I ; P |
| DIHYDLIPOXN-RXN | I ; P |
| GLU6PDEHYDROG-RXN | I |
| GLUCOKIN-RXN | I |
| GLUCOSE-6-PHOSPHATE-1-EPIMERASE-RXN | I |
| PYRUVDEH-RXN | I ; P |
| RXN0-6478 | G ; I |
| RXN0-6485 | G ; I |
| RXN-10 | T |
| RXN-10639 | Sp ; I |
| RXN-12496 | I |
| RXN-12496 | G ; I ; T |
| RXN-12753 | Sp ; I |
| RXN-12754 | Sp ; I |
| RXN-3341 | I |
| RXN-4821 | Sp ; I |
| RXN-8141 | G ; I |
| RXN-9 | T |
| RXN-9952 | I |

**Table S5 : Reactions in the metabolic network of *Portiera*.** Legend: G(eneric reactions), I(solated reactions), P(seudogenes), Sp(ontaneous reactions), T(axonomic issues).

| **Reaction** | **Reasons** |
| --- | --- |
| DIAMACTRANS-RXN | G ; I |
| RXNQT-4142 | I |
| RXN-11836 | G ; I |
| TYRAMINOTRANS-RXN | I |
| RXN-9939 | I |
| 5.3.3.14-RXN | G ; I |
| ADOMET-DMK-METHYLTRANSFER-RXN | I |
| SUCCGLUALDDEHYD-RXN | I |
| RXN0-5305 | I ; Sp |
| RFFTRANS-RXN | I |
| 3.6.3.2-RXN | I |
| RXN-12149 | I ; Sp |
| RXN0-1241 | G ; I |
| 3.6.3.39-RXN | I |
| PEPTIDYLPROLYL-ISOMERASE-RXN | G ; I |
| PROPKIN-RXN | I |
| DIHYDROPYRIMIDINASE-RXN | I |
| BETA-LACTAMASE-RXN | G ; I |
| RXN0-5408 | I |
| CHOLINE-KINASE-RXN | I |
| AQUACOBALAMIN-REDUCTASE-RXN | G ; I |
| RXN-11837 | I |
| RXN-11838 | I |
| RXN-11839 | I |
| RXN-4122 | G ; I ; Sp |
| 3.1.22.4-RXN | I |
| ALDOSE-1-EPIMERASE-RXN | X |
| RXN-11833 | I |
| RXN0-5100 | I |
| DSBBPROT-RXN | I |
| RXN0-5131 | G ; I |
| HYDROXYPYRROLINEDEH-RXN | I |
| HYDROXYPRODEHYDROG-RXN | I |
| RXN-10720 | I ; Sp |
| 2.6.1.7-RXN | I |
| 5.99.1.3-RXN | G ; I |
| 5.99.1.2-RXN | G ; I |
| 3.4.19.12-RXN | G ; I |
| RXN0-5190 | G ; I |
| 3.2.1.17-RXN | G ; I |
| 3.4.23.36-RXN | G ; I |
| RXN0-5195 | I |
| RXN0-3182 | I |
| 3.4.11.2-RXN | I |
| RXN0-5225 | I |
| CARBOXYLESTERASE-RXN | G ; I |
| 1.11.1.15-RXN | I |
| RXN-10015 | I ; Sp |
| RXN-10017 | I ; Sp |
| RXN-10016 | I ; Sp |
| RXN-10018 | I ; Sp |
| DIHYDLIPOXN-RXN | P |
| DIHYDLIPACETRANS-RXN | P |
| RXN0-5388 | G ; I |
| RXN-12508 | G |
| 3.5.1.88-RXN | G ; I |
| NADPH-DEHYDROGENASE-FLAVIN-RXN | I |
| RXN-12445 | I |
| PMPOXI-RXN | I |
| RXN-2961 | I ; Sp |
| RXN0-6564 | I |
| SPONTPRO-RXN | Sp |
| RXN0-4461 | I |
| RXN0-5515 | I |
| TEICHOICSYN4-RXN | I |
| RXN-12726 | I |
| THI-P-KIN-RXN | I |
| 3.6.1.41-RXN | I |
| TRANS-RXN-167A | I |
| TRANS-RXN-168 | I |
| RXN0-17 | I |

**Table S6: Necessary cofactors for the *Portiera* enzymes.** The left column gives the list of the cofactors (based on Uniprot information), the right column the number of associated enzymes.

| **Cofactor** | **Enzymes** |
| --- | --- |
| Mg | 11 |
| Zn | 9 |
| Fe | 11 |
| Mn | 4 |
| Co | 2 |
| Cu | 1 |
| Na | 1 |
| K | 1 |
| Pyridoxal phosphate | 7 |
| FAD | 4 |
| Thiamine | 2 |
| Lipoyl | 2 |
| Protoheme | 1 |
| NAD | 1 |
| FMN | 1 |
